# Cardiovascular risk associated with high sodium-containing drugs: A systematic review

**DOI:** 10.1371/journal.pone.0180634

**Published:** 2017-07-06

**Authors:** Germain Perrin, Virginie Korb-Savoldelli, Alexandre Karras, Nicolas Danchin, Pierre Durieux, Brigitte Sabatier

**Affiliations:** 1 Department of Pharmacy, Georges Pompidou European Hospital, Paris, France; 2 INSERM UMR 1138 Team 22: Information Sciences to Support Personalized Medicine, Paris Descartes University, Paris, France; 3 Faculty of Pharmacy, Clinical Pharmacy Department, Paris Saclay University, Châtenay-Malabry, France; 4 Department of Nephrology, Georges Pompidou European Hospital, Paris, France; 5 Paris Descartes University, Paris, France; 6 Department of Cardiology, Georges Pompidou European Hospital, Paris, France; 7 Department of Biomedical Informatics and Public Health, Georges Pompidou European Hospital, Paris, France; The University of Tokyo, JAPAN

## Abstract

**Background:**

Excess dietary sodium is associated with increased blood pressure (BP). Some drugs are associated with high sodium intake (in particular effervescent tablets), but the cardiovascular risk associated with such high sodium-containing drugs (HSCD) is largely underevaluated.

**Objectives:**

To summarize the evidence for a potential cardiovascular risk associated with exposure to HSCD, and to highlight possible risk factors associated with this iatrogenic issue; in general and/or specific populations.

**Methods:**

We conducted a systematic review, by searching electronic databases including MEDLINE, EMBASE, Web of Science, CENTRAL and grey literature between 1960 and 2015. We included studies that reported modification of cardiovascular parameters or incidence/prevalence of cardiovascular outcomes, between a group of subjects exposed to HSCD relative to a non-exposed group. The threshold used to identify HSCD was 391 mg/day. We did not consider studies evaluating exposure to sodium as an active ingredient or those focusing on dialysis solutions or enteral/parenteral nutrition. Study quality was assessed using the EPHPP tool.

**Results:**

A total of eight studies met our inclusion criteria. Four reported results for short-term exposure to HSCD (≤ 7 days) on BP fluctuations. One study reported an elevation of BP (associated sodium intake: 1,656 mg/day). Four studies evaluated a long-term exposure (≥ 2 years or discontinuation of a chronic treatment). Two studies reported iatrogenic risk. For these studies, drug associated sodium intake was high (> 1,500 mg/day) in patients with comorbidities (in particular, diabetes mellitus and hypertension).

**Conclusion:**

Despite numerous study limitations, this systematic review suggests three potential synergistic risk factors for cardiovascular complications after exposure to HSCD: a high sodium intake (≥ 1,500 mg/day), a long duration of exposure, and the presence of comorbidities. Further studies are required to characterize this iatrogenic risk.

**Trial registration:**

PROSPERO CRD42016047086.

## Introduction

The relationship between high dietary sodium intake and hypertension is well established [1–4], but there is currently insufficient evidence of a direct association between sodium intake and mortality and/or cardiovascular outcomes [3,5,6]. The WHO recommends limiting daily salt consumption in the general population to 5 g per day, which is equivalent to 2 g of sodium [4]. The relevance of this population-based approach has been the subject of debate, in part because not all subjects exhibit changes in blood pressure parallel to changes in salt intake [7]. Indeed, several environmental and demographic factors are associated with the salt-sensitive blood pressure (SSBP) phenotype (i.e. increased blood pressure associated with increased salt intake): sex (women are more salt-sensitive), greater age, obesity, black ethnicity, and clinical conditions, including pre-existing hypertension, chronic kidney disease, and diabetes mellitus [8,9].

Although the main source of sodium intake is associated with food (especially bread, cheese, and processed food), other potentially important sources are often overlooked. For example, sodium is widely used in drug formulation. It may be used as an active ingredient (for physiological sodium replacement) or as a cation associated with an excipient (such as sodium bicarbonate or sodium citrate). Excipients containing sodium fulfil multiple roles: for example, lubrication, disintegration, solubility enhancement, or alkalization. Some pharmaceutical formulations contain large amounts of sodium, such as effervescent tablets, alginates, or intravenous antibiotics. These high sodium-containing drugs (HSCD) can significantly contribute to global sodium intake and could theoretically provoke or worsen cardiovascular conditions, particularly hypertension. An observational French study, published in 2005, assessed exposure to HSCD in 137,910 patients chronically treated with diuretics (analysis based on a medico-administrative database): over a period of six months, 39% of patients were exposed to HSCD, of whom 26% were frequently/very frequently exposed. In total, 9% of patients received a cumulative average daily dose of more than 0.8 g sodium during the six-month period (i.e. 40% of the WHO's recommended daily dose) [10]. A study published by George *et al*. in 2013 showed that patients diagnosed with hypertension or cardiovascular events (non-fatal stroke, myocardial infarction, or vascular death) were more likely to be prescribed HSCD [11]. In 2015, the European Medicines Agency (EMA) published an update of its 2003 guideline 'Excipients in the label and package leaflet of medicinal products for human use', focusing on sodium [12]. This revision aimed to provide readily available information to healthcare professionals and patients about the sodium content of drugs, via drug packages and/or leaflets. This revision proposed a threshold of 391 mg/day (approximately 20% of the WHO adult recommended maximum daily dietary intake for sodium) for considering the drug to be "high sodium". Despite these guidelines, based on the precautionary principle, little is known about the clinical impact of exposure to HSCD, and its determinants (existence of high risk populations, importance of the nature of associated anion etc.).

The aim of the present systematic review was to summarize the evidence for the under-evaluated and underestimated issue of iatrogenic risk associated with HSCD exposure, in general and/or specific populations; and to highlight possible risk factors for developing such iatrogenic complications.

## Methods

This systematic review is presented according to the *Preferred Reporting Items for Systematic Reviews and Meta-analyses* (PRISMA) guidelines [13]. The study protocol has been registered in the PROSPERO database (registration number: CRD42016047086).

### Data sources and search strategy

We identified potentially eligible studies by searching MEDLINE, using the following MeSH terms: 'sodium', 'cardiovascular diseases', 'pharmaceutical preparations', and 'drug therapy'. Our search was restricted to human studies, published in English or French, between 1960 and 2015. We also searched EMBASE using a similar search strategy. The other databases used were Web of Science and CENTRAL. All search strategies are shown in S1 Fig. The electronic search was run on June 5, 2016.

Two authors independently screened all retrieved citations based on the titles and abstracts. Study eligibility was assessed by reading the full text. Agreement between the two reviewing authors was assessed using Cohen's κ statistic [14]. Disagreements were resolved by discussion and consensus.

### Study selection criteria

We systematically identified peer-reviewed original studies, which quantitatively reported modifications of a cardiovascular parameter or incidence/prevalence of a cardiovascular outcome, or a modification of a cardiovascular therapy, between a group of subjects exposed to HSCD relative to a non-exposed group. Drugs of interest were those which contained sodium as a cation of an excipient, or of an active ingredient (only drugs which brought 391 mg sodium or higher in the daily dose were considered as HSCD and were then included). Studies for which sodium was the active ingredient were not considered in the review. Studies focusing on the electrolytic composition of dialysis solutions, a specific topic which has already been reviewed elsewhere [15], or enteral/parenteral nutrition were also excluded from the present review. The study designs of interest included: experimental studies (randomized controlled trials) and quasi-experimental studies (non-randomized controlled trials, controlled or non-controlled before-after studies and interrupted time series), observational studies (cross-sectional, retrospective/prospective cohort, or case-control studies). Reviews, letters, commentaries/editorials, case series, and case reports were not included in the review.

### Data extraction and collection

Data extraction was independently performed using a standardized data extraction form by two authors. General data extracted from full-text included: year of publication, country, study design, number of centres, number of patients, mean age, percentage of women, prevalence of comorbidities in the baseline characteristics (including diabetes mellitus, hypertension, and chronic kidney disease), percentage of black patients, and percentage of patients treated with renin-angiotensin system blockers. Other data extracted were those related to HSCD: type of sodium salt, amount of sodium per day of treatment, route of administration, duration of exposure to the HSCD, and the medical indication. Extracted outcomes data included: nature of the outcome, data collection method, and the quantitative result associated with the outcome. Corresponding authors were contacted for additional information, if necessary.

### Quality assessment of included studies

Assessment of the study quality was performed independently by two authors using the validated Effective Public Health Practice Project (EPHPP) quality assessment tool [16]. This instrument covers any quantitative study design (randomized or not, controlled or not), and is based on the evaluation of six types of bias: selection bias, study design, confounders, blinding, data collection method, and withdrawals and dropouts. An overall evaluation was performed and a strong rating assigned to a study if there was no weak component score. A moderate rating was assigned if there was only one weak component score, and a weak rating if there were two or more weak component scores.

## Results

### Results of the search strategy and general study characteristics

**Fig 1:**
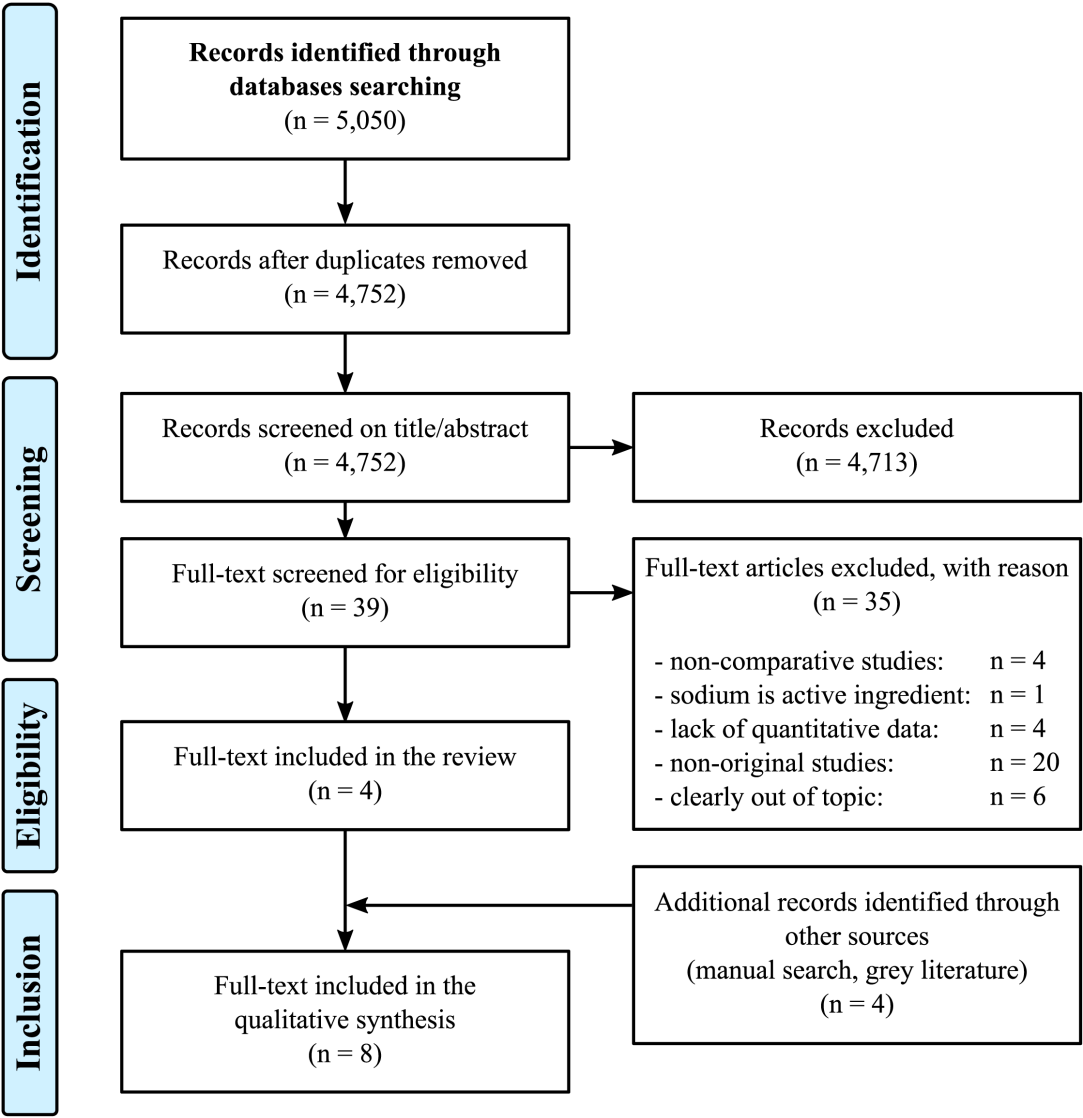
PRISMA flowchart summarizing the identification and selection process of the studies for inclusion in the systematic review.

We identified 5,050 references, resulting in 4,752 unique citations, after removal of duplicates (298). A flow diagram summarizing the identification and selection process for included studies is presented in Fig 1.

We screened 4,752 titles and abstracts, and excluded 4,713 irrelevant publications. A total of 39 publications were reviewed for eligibility. Among these, 35 were excluded for the following reasons: the study was not comparative, sodium was the active ingredient (one study), lack of quantitative data, or the study was not original or out of topic. Four studies finally met the inclusion criteria [11,17–19]. We found four additional records through other sources, including grey literature, by manual research [20–23]. Thus, a total of eight studies were included in the present analysis. Agreement between the reviewers was high, with a kappa coefficient of 0.87 [95% CI: 0.63–1.1].

The characteristics of the studies included in the review are summarized in Table 1.

**Table 1:** General characteristics of the eight studies included in the systematic review.

Authors	Year	Country	Design	Centres (n)	Subject (n)	Original purpose	Intervention medication (HSCD)	Control medication	Reported outcome	Duration of exposure to HSCD
Husted *et al*. [20]	1975	USA	uBA	1	10	safety of oral NaHCO_3_ in CKD	sodium bicarbonate	sodium chloride	BP fluctuations	4 days
Mark *et al*. [17]	1993	USA	uBA	1	40	safety of intraoperative intraveinous NaHCO_3_ therapy	sodium bicarbonate	sodium chloride	BP fluctuations	1 day
Passfall *et al*. [21]	1997	Germany	RCTx	1	11	safety of water and NaHCO_3_ loading in CDK	sodium bicarbonate	control water	BP fluctuations	7 days
de Brito-Ashurst *et al*. [22]	2009	UK	RCTp	1	134	safety of oral NaHCO_3_ in CKD	sodium bicarbonate	no medication	BP fluctuation, hospitalization for CHF, worsening hypertension or oedema	2 years
Ubeda et al. [18]	2009	Spain	uBA	2	34	safety of effervescent drugs in hypertensive patients	effervescent paracetamol	non effervescent paracetamol	BP fluctuations	≥ 4 weeks*
Mahajan et al. [23]	2010	USA	RCTp	1	40	safety of oral NaHCO_3_ in early hypertensive nephropathy	sodium bicarbonate	sodium chloride and placebo	BP fluctuations	5 years
George et al. [11]	2013	UK	nCC	na	122,144	cardiovascular safety of HSCD	variable	Corresponding standard formulations of the selected HSCD	BP fluctuations, stroke, myocardial infarction, heart failure, all cause mortality, vascular death	3.92 years
Thomas *et al*. [19]	2014	UK	RCTp	1	110	safety of oral sodium alginate + NaHCO_3_	sodium bicarbonate + sodium alginate	placebo	BP fluctuations	7 days

BP: blood pressure, CDK: chronic kidney disease, CHF: congestive heart failure, HSCD: high sodium-containing drug, na: not applicable, nCC: nested case-control study, RCTp: randomized controlled trials with parallel groups, RCTx: randomized controlled trial with crossover design, uBA: uncontrolled before-after studies.

* duration of follow up after discontinuation of a chronic treatment with effervescent paracetamol.

The time span of publication is wide (between 1975 and 2014). The studies varied widely in population size (ranging from 10 to 122,144 for the nested case-control study). Most of the studies were single-centre (6/8). The study design also varied, half were randomized controlled trials [19,21–23], three were uncontrolled before-after studies [17,18,20], and one was a nested case-control study [11]. Baseline cardiovascular and renal functions, and exclusion criteria are given in Table 2.

**Table 2:** Baseline cardiovascular and renal functions, before patient exposure to HSCD, and exclusion criteria, in particular those associated with these functions.

	Husted	Mark	Passfall	de Brito-Ashurst	Ubeda	Mahajan	George	Thomas
	1977	1993	1997	2009	2009	2010	2013	2014
**Cardiovascular function**								
- Hypertension* (%)	nm	47.5	nm	27.5	100	100	nm	nm
- SBP (mmHg)	nm	nm	118 ± 19	124 ± 1.3	159.6 ± 3.3	155.3 ± 12.6	nm	nm
- DBP (mmHg)	nm	nm	nm	76.1 ± 1.5	94.4 ± 1.7	nm	nm	nm
- CHF* (%)	nm	15	nm	0	nm	0	7	nm
**Renal function**								
- CDK* (%)	100	nm	nm	100	nm	100	8	nm
- EGFR (mL/min)	10.5 ± 4.5	nm	12.7 ± 15.9	20.1 ± 6.5	nm	73.2 ± 6.0	nm	nm
**Cardiovascular drugs**	nm	nm/no drug with hemodynamic effects	nm/no discontinuation of chronic treatments, including diuretics	Loop diuretics (70%), moxonodine (17%), α-blockers (59%), β-blockers (19%), Ca-blockers (50%), RAS-blockers (50%)	RAS-blockers (55.9%), diuretics (26.5%), Ca- blockers (20.6%), β-blockers (11.8%)	nm/RAS-blockers (100%)	Cardiovascular drugs (66%), including RAS-blockers (32%)	nm
**Exclusion criteria**	**Poorly controlled BP**; unstable clinical state; presence of oedema	Need for drugs that might affect hemodynamic status	**Decompensated CHF; poorly controlled angora pectoris**, presence of valvular lesions	**Poorly controlled BP; CHF**; malignant disease, morbid obesity, cognitive impairment, chronic sepsis	nm	**Primary kidney disease; clinical evidence of cardiovascular disease; peripheral oedema**; tobacco use, drug non-compliance, diabetes, malignancy intolerance to RAS-blockers	Malignancy, salt wasting conditions, malabsorption, history of substance abuse	**Disease requiring low Na diet;** any severe disease; concomitant treatment with proton pump inhibitors, anti-H_2_, prokinetics, systemic glucocorticoids or anti-inflammatory drugs, pregnant or lactating women

BP: blood pressure; CDK: chronic kidney disease, CHF: congestive heart failure; DBP: diastolic blood pressure; EGFR: estimated glomerular filtration rate; nm: not mentioned; RAS: renin-angiotensin system; SBP: systolic blood pressure. Data are expressed as the mean ± SD.

* No details about disease severity: clinical diagnosis only.

### Study characteristics associated with the SSBP phenotype

We extracted the population characteristics associated with the SSBP phenotype from the eight studies (Table 3). All studies included an adult population (mean age: 43–68.2). The proportion of women varied from 0 to 59%. The proportion of hypertensive patients varied from 0% in one study (hypertension was an exclusion criterion) to 100% in two studies (hypertension was an inclusion criterion). The proportion of diabetic patients was available for four studies [11,21–23] and varied from 0% (exclusion criterion) to 36.5%. The percentage of patients with chronic kidney disease varied from 0% in one study (exclusion criterion) to 100% in four studies (inclusion criterion). The proportion of obese patients was available for only one study and was 17%. The proportion of patients of black ethnicity varied from 0% in one study to 63% in another, and was not specified in six. The percentage of patients treated with renin-angiotensin system blockers ranged from 36 to 100% (inclusion criterion).

**Table 3:** Characteristics of study populations associated with SSBP phenotype.

Authors	Mean age (year)	Women (%)	Hypertension (%)	CKD* (%)	Diabetes mellitus (%)	Treatmentwith RAS blockers (%)	Obesity** (%)	Black ethnicity (%)
Husted *et al*. [20]	**Y** (49.8)	**Y** (40)	**N**	**Y** (100)	**N**	**N**	**N**	**N**
Mark *et al*. [17]	**Y** (64.5)	**Y** (0.0)	**Y** (47.5)	**N**	**N**	**N**	**N**	**N**
Passfall *et al*. [21]	**Y** (61.7)	**Y** (45)	**Y** (27)	**Y** (100)	**Y** (18)	**N**	**N**	**N**
de Brito-Ashurst *et al*. [22]	**Y** (54.8)	**Y** (48.5)	**Y** (27.5)	**Y** (100)	**Y** (36.5)	**N**	**N**	**N**
Ubeda *et al*. [18]	**Y** (68.2)	**Y** (38.2)	**Y** (100)	**N**	**N**	**Y** (55.9)	**N**	**N**
Mahajan *et al*. [23]	**Y** (51.3)	**Y** (52)	**Y** (100)	**Y** (100)	**Y** (0.0)	**Y** (100)	**N**	**N**
George *et al*. [11]	**Y** (69)	**Y** (59)	**N**	**Y** (8.6)	**Y** (16)	**Y** (36)	**Y** (17)	**N**
Thomas *et al*. [19]	**Y** (43)	**Y** (45.5)	**Y** (0.0)	**Y** (0.0)	**N**	**N**	**N**	**Y** (0.0)

CKD: chronic kidney disease, ns: not specified, RAS: renin-angiotensin system. **Y**: item reported in the publication (proportion of the study population presenting the characteristic); **N**: item not reported in the publication

* defined by an estimated glomerular filtration rate < 60 mL/min

** defined by a body mass index > 25 kg.m^-2^.

### Study characteristics associated with HSCD exposure

Data concerning drug-associated sodium intake are given in Table 4. Most of the studies evaluated the cardiovascular and/or pharmacotherapeutic impact of exposure to sodium bicarbonate (associated or not with other sodium salts, such as sodium citrate or sodium alginate). Seven studies evaluated oral exposure, whereas only one study evaluated intravenous exposure to sodium bicarbonate as a single bolus [17]. The therapeutic indications were: nephroprotection in patients with chronic kidney disease (3/8), correction of metabolic acidosis (2/8), pain relief (2/8), and gastro-oesophageal reflux (1/8). The sodium intake per day associated with these drugs varied from 464 to 4,623 mg. The duration of exposure allowed us to differentiate two kinds of exposure: short-term exposure (≤ 7 days; four studies), and long-term exposure (≥ 2 years or chronic treatment; four studies).

**Table 4:** Description of the drug-associated sodium intake.

Authors	Drug	Route	Posology	Therapeutic indication	Type of Na salt	Drug associated Na intake (/day in mg)	Duration of exposure to HSCD
Husted *et al*. [20]	sodium bicarbonate	po	1,688 mg per day	metabolic acidosis	sodium bicarbonate	4,623	4 days
Mark *et al*. [17]	sodium bicarbonate	iv	single bolus [3,444–7,392 mg]	metabolic acidosis	sodium bicarbonate	1,656 [943–2,24]	1 day
Passfall *et al*. [21]	sodium bicarbonate	po	2L solution per day	nephroprotection	sodium bicarbonate	1,840	7 days
de Brito-Ashurst *et al*. [22]	sodium bicarbonate	po	600 mg three time a day*	nephroprotection	sodium bicarbonate	493	2 years
Ubeda *et al*. [18]	paracetamol + sodium bicarbonate + sodium citrate	po	1,000 mg paracetamol three time a day	pain relief	sodium bicarbonate + sodium citrate	1,700	Discontinuation of a chronic treatment
Mahajan *et al*. [23]	sodium bicarbonate	po	11.5 mg/kg/day (tablets)	nephroprotection	sodium bicarbonate	805 (adult of 70 kg)	5 years
George *et al*. [11]	list of 24 HSCD	po	variable	variable	variable	2,438	3.92 years
Thomas *et al*. [19]	sodium alginate + sodium bicarbonate + calcium carbonate	po	8 tablets per day	gastroesophageal reflux	sodium bicarbonate + sodium alginate	464	7 days

HSCD: high sodium-containing drug, iv: intravenous, po: per os.

* Dose increased as necessary to maintain HCO3^-^ levels ≥ 23 mmol.L^-1^.

### Results of short-term studies evaluating only BP fluctuations

The results for short-term studies are given in Table 5. These studies all evaluated blood pressure fluctuations as a surrogate marker for cardiovascular outcomes. Three studies did not find a significant effect of oral HSCD exposure on blood pressure [19–21]. The drug-associated sodium intake varied widely (ranging from 464 to 4623 mg/day). These studies all excluded patients with clinical conditions associated with a low sodium diet requirement. The study of Mark *et al*. [17] evaluated the safety of a single intravenous bolus of sodium bicarbonate in patients with coronary disease who developed intraoperative metabolic acidosis. They found an isolated and significant elevation of both systolic (SBP) and diastolic blood pressure (DBP) in the NaHCO_3_ group between T_0_ and T_5min_ (from 118 ± 19 to 130 ± 26 mmHg, p < 0.05), which was independent of the volume load (no significant difference between T_0_ and T_5min_ in either DBP or SBP in the control group which received NaCl, with equivalent sodium and volume loading conditions between both groups).

**Table 5:** Results of studies evaluating the effect of short-term exposure to HSCD (≤ 7 days) on cardiovascular events.

Authors, year, n	Outcome	Outcome evaluation method	Blinding process	Results
Husted *et al*. [20], 1975, n = 10	SBP fluctuations	2 measures after a 15 min rest period	nd	Increase of 15.5 ± 6.7 mmHg in the NaCl group vs decrease of 4.0 ± 3 mmHg in the NaHCO_3_ group between day 1 and day 5 (p < 0.01)
Mark *et al*. [17], 1993, n = 40	SBP/DBP fluctuations	Cardiopulmonary catheterization	Double	Increase of SBP from 118 ± 19 to 130± 26 mmHg in the NaHCO_3_ group between T_0_ and T_5min_ (p < 0.05)
				No DBP changes in the NaHCO_3_ group between T_0_ and T_5min_ (no quantitative data)
Passfall *et al*. [21], 1997, n = 11	SBP/DBP fluctuations	Sphygmomanometric measure (not detailed)	Double	No SBP changes between day 0 and day 7 (136.5 ± 29 vs 138 ± 29.5 mmHg between control and NaHCO_3_ groups, respectively, p > 0.05)
				No DBP changes between day 0 and day 7 (79 ± 21 vs 77 ± 21 mmHg between control and NaHCO_3_ groups, respectively, p > 0.05)
Thomas *et al*. [19], 2014, n = 110	BP fluctuations (no details)	Not specified	Double	1.8% increase of BP in treatment group vs 1.9% in the control group (p = 1)

*BP*: *blood pressure*, *CHF*: *congestive heart failure*, *DBP*: *diastolic blood pressure*, *HSCD*: *high sodium- containing drugs*, *nd*: *not described*, *SBP*: *systolic blood pressure*. Data are expressed as the mean ± SD.

### Results of long-term studies evaluating only BP fluctuations

The results of long-term exposure are given in Table 6. Mahajan *et al*. [23] studied the effect of a five-year exposure to sodium bicarbonate as a nephroprotectant in 40 patients with early hypertensive nephropathy. The drug-associated sodium intake was 805 mg/day. They found no significant effect on SBP after five years: 135.1 ± 6.2 vs 133 ± 8.1 mmHg for the bicarbonate and placebo groups, respectively (overall p = 0.179).

**Table 6:** Results of studies evaluating the effect of long-term exposure to HSCD *(≥ 2 years*) or the effect of discontinuation of a chronic treatment with HSCD on cardiovascular events.

Authors, year, n	Outcome	Outcome evaluation method	Blinding process	Results
**LONG-TERM EXPOSURE *(≥ 2 years* days)**				
de Brito-Ashurst *et al*. [22], 2009, n = 134	SBP/DBP fluctuations	During standard clinical management (not specified)	Simple	Percentage of patients with BP > 130/80 mmHg (control vs NaHCO_3_): 10 vs 14% at T_0_, 15 vs 16% at year 1, 16 vs 17% at year 2 (overall p = 0.6)
	Hospitalization for decompensated CHF			0% (control group) vs 0% (NaHCO_3_ group
	Worsening hypertension requiring more intensive therapy			48% (control group) vs 61% (NaHCO_3_ group), p = 0.17
	Worsening oedema requiring increase in loop diuretics			30% (control group) vs 39% (NaHCO_3_ group), p = 0.5
Mahajan *et al*. [23], 2010, n = 40	SBP fluctuations	Not specified	Simple	After 5 years, variation of SBP between NaHCO_3_, placebo, and NaCl groups: 135.1 ± 6.2, 133 ± 8.1 mmHg and 132.1 ± 6.6, respectively (overall p = 0.179)
George *et al*. [11], 2013, n = 122,144	Composite outcome*	Measurement at the general practitioner's office (not specified)	na	OR: 1.16 [95% CI: 1.12–1.21]
	Hypertension			OR: 7.18 [95% CI: 6.74–7.64]
	Incident non-fatal stroke			OR: 1.22 [95% CI: 1.16–1.29]
	All-cause mortality			OR: 1.28 [95% CI: 1.23–1.33]
	Incident non-fatal myocardial infarction			OR:0.94 [95% CI: 0.88–1]
`	Vascular death			OR: 0.7 (95% CI: 0.31–1.59]
	Heart failure			OR: 0.98 [95% CI: 0.93–1.04]
**DISCONTINUATION OF A CHRONIC TREATMENT**				
Ubeda *et al*. [18],2009, n = 34	SBP/DBP fluctuations	3 measurements by a trained community pharmacist (not specified)	nd	Decrease of SBP of 13.1 mmHg [95% CI: 11.9–14.3, p < 0.0001], and decrease of DBP of 2.5 mmHg [95% CI: 2.1–2.9, p < 0.0001] after discontinuation of chronic treatment with effervescent paracetamol.

*BP*: *blood pressure*, *CHF*: *congestive heart failure*, *DBP*: *diastolic blood pressure*, *HSCD*: *high sodium- containing drugs*, *na*: *not applicable*, *nd*: *no data*, *ns*: *not significant*, *SBP*: *systolic blood pressure*. Data are expressed as the mean ± SD.

* composite outcome: incident non-fatal MI, incident non-fatal stroke, vascular death.

### Results of long-term studies evaluating BP fluctuations and cardiovascular outcomes

De Brito-Ashurst *et al*. [22] studied the impact of chronic exposure (two years) to oral sodium bicarbonate on BP fluctuations, admission rates for congestive heart failure, worsening hypertension requiring more intensive therapy, and worsening oedema requiring increased use of loop diuretics in patients with chronic kidney disease. They found no significant impact of chronic exposure on any of these parameters. The study of George *et al*. [11] was a nested case-control study, based on the UK Clinical Practice Research Datalink (CRPD) clinical database. The study population was comprised of 1,292,337 patients over the age of 18 years. The authors found that patients who survived a stroke, myocardial infarction, or who died of a cardiovascular condition had a higher risk of being prescribed an effervescent, soluble, or dispersible medicine (considered to be an HSCD) than patients who did not experience one of these events (odds ratio 1.16 [95% CI: 1.12–1.21]). They also found that hypertensive patients had an odds ratio of 7.18 [95% CI: 6.74–7.64] for being prescribed a HSCD relative to the control group. The odds ratio was 1.22 [95% CI: 1.16–1.29] for patients who survived a stroke. The odds ratio for all-cause mortality also increased (OR: 1.28 [95% CI: 1.23–1.33]). They found no association with incident non-fatal myocardial infarction, heart failure, or vascular death.

### Results of a study evaluating discontinuation of chronic exposure to HSCD on only BP fluctuations

Ubeda *et al*. [18] studied the effect of discontinuation of chronic treatment with effervescent paracetamol in 34 elderly patients with osteoarthritis pain and uncontrolled BP (Table 6). All were treated with renin-angiotensin blockers. They found that switching from the effervescent medication to the standard formulation (considered to be sodium free) was associated with a large and significant decrease in both SBP and DBP (-13.1 mmHg for SBP [95% CI: 11.9–14.3, p < 0.0001], and -2.5 mmHg for DBP [95% CI: 2.1–2.9, p < 0.0001]). The median interval between discontinuation and BP measurement was 58 days.

### Quality assessment of included studies

The intra-study risk of bias assessment using the EPHPP tool is presented in Table 7. Most of the studies had a high global risk of bias (6/8). Two studies had a moderate global risk of bias [11,23], and no study had a low risk of bias. The main risks of bias found in the included studies are summarized in S2 Fig. More than half of the studies had important issues with selection bias (monocentric studies, self-referred patients etc.) and confounders that were insufficiently taken into account (classical confounders associated with cardiovascular risk, confounders associated with the SSBP trait, and other confounders associated with patient medications). Additionally, four studies had important issues with the data collection process that was insufficiently described (for example, no information concerning the modalities used for blood pressure assessment).

**Table 7:** Study quality. Assessment of the intra-study risk of bias using the EPHPP tool.

Type of bias	Husted	Mark	Passfal	de Brito-Ashurst	Ubeda	Mahajan	George	Thomas
	1977	1993	1997	2009	2009	2010	2013	2014
***Selection***	**H**	**H**	**H**	**M**	**H**	**L**	**L**	**H**
***Design***	**L**	**H**	**L**	**L**	**H**	**L**	**M**	**L**
***Confounders***	**L**	**H**	**L**	**H**	**H**	**M**	**H**	**H**
***Blinding***	**M**	**L**	**L**	**M**	**H**	**M**	N.A	**L**
***Data collection***	**H**	**M**	**M**	**H**	**L**	**H**	**L**	**H**
***Withdrawals and dropouts***	**L**	**L**	**H**	**L**	**M**	**L**	N.A	**L**
**Global risk**	**H**	**H**	**H**	**H**	**H**	**M**	**M**	**H**

**L:** Low risk of bias; **M:** moderate risk of bias; **H:** high risk of bias; N.A: not applicable

## Discussion

The present review highlights the lack of data regarding the possible iatrogenic risk associated with exposure to HSCD, with a globally low level of evidence. Only eight studies were included, of which only two were specifically designed to assess this issue. Randomized controlled trials were limited by the size of the population, and other studies were limited by methodological weaknesses. However, this review highlights three possible expected risks factors, probably acting synergistically.

### Search strategy and study quality

We identified only eight studies which have assessed the cardiovascular consequences associated with exposure to HSCD. Among them, five were randomized controlled trials, with a generally small sample size (10 to 134 subjects). For ethical reasons (it was not possible to expose high risk patients to HSCD), Thomas *et al*. [19] excluded patients with hypertension and/or chronic kidney disease, which are important demographic factors associated with SSBP [9]. This may have limited the power of the trial to detect a possible iatrogenic consequence of exposure to HSCD. Two trials were uncontrolled before-after studies (20,23), which is a study design associated with a very low level of evidence and a high risk of bias. This design is not recommended by Cochrane [24]. Most trials were monocentric studies, introducing a high risk of selection bias. Only one study had two inclusion centres [18]. The last study was a nested case-control study which used the CRPD database [11]. This database aggregates information from more than 6.5% of the UK population, and is considered to be representative of the general UK population [25].

Generally, confounders were poorly taken into account due to the large number of such covariates: classical cardiovascular risk factors (diabetes mellitus, hypertension, etc.), risk factors associated with SSBP (black ethnicity, obesity, genetic variations, etc.), and confounders associated with concomitant treatments and comorbidities. Some drugs interfere with the control of hypertension, including corticosteroids, non-steroidal anti-inflammatory drugs, immunosuppressant, and oestrogen and progestin. [26]. For other drugs, such as paracetamol, there is evidence suggesting an association between exposure and an elevation of blood pressure [27], but this is not corroborated by other studies [28,29]. Chronic pain may also be associated with a higher prevalence of hypertension [30]. These last two points are crucial because effervescent paracetamol, one of the most popular HSCD, is widely prescribed and available over-the-counter, resulting in a large number of exposed subjects, sometimes chronically. Studies evaluating the effect of effervescent paracetamol should also take into account pain intensity as a covariable.

The crossover design may partially resolve the issue associated with the multiplicity of confounders. This is due to the ability of crossover to control confounders with a fixed effect over time, such as ethnicity or genetic variations (these confounders are generally poorly or not controlled in randomized controlled trials with parallel groups or in case-control studies).

Results from the two crossover studies [20,21] in this review should be interpreted cautiously because of a possible carryover effect. Indeed, the washout period between sodium salt supplementation and control periods appeared to be too short (two to three days in the study of Husted *et al*. and three days in the study of Passfall *et al*.). Generally, sodium balance is achieved in three to five days [31] in healthy subjects with preserved kidney and cardiac functions, and populations from these two studies included patients with advanced chronic kidney failure.

Our search strategy identified a protocol for a randomized controlled trial published in 2015, which is still recruiting patients, which will evaluate the relationship between effervescent paracetamol (as a HSCD) and blood pressure [32]. This crossover trial is recruiting 49 hypertensive patients > 18 years of age with controlled hypertension. The primary endpoint will be variations in SBP assessed by 24-hour ambulatory blood pressure monitoring. Exposure to HSCD will be three weeks. Exclusion criteria include: an estimated glomerular filtration rate < 30 mL/min, poorly controlled hypertension, diagnostic of heart failure, and a history of cardiovascular events during the last six months. However, the relatively short-term exposure to HSCD and exclusion of patients with a high risk of sodium overload may limit the power of this study to detect any iatrogenic alert.

### Risk factors associated with HSCD exposure-dependent cardiovascular iatrogenic risk

We compared the characteristics of positive (i.e. showing an iatrogenic risk) and negative studies (i.e. showing a favourable safety profile) with expected risk factors for the development of sodium and water overload associated with high dietary sodium intake, despite the low level of evidence provided by studies included in this systematic review. This comparison allows us to suggest three potential synergistic risk factors associated with cardiovascular complications after exposure to HSCD:

1. A daily sodium intake associated with HSCD > 1500 mg.
2. Long-term exposure to HSCD. Indeed, the risk of sodium and water overload, and resulting hypertension, is higher when high salt intake is maintained over a long period of time, probably by a mechanism involving end-stage damage due to hypertension, in particular kidney injury [33].
3. The presence of comorbidities: particularly hypertension and diabetes mellitus.

Surprisingly, no obvious iatrogenic risk was found in the two long-term studies of patients with chronic kidney disease [22,23]. Possible explanations include: (i) the included patients may have been advised to limit their salt intake associated with food in the context of their chronic kidney disease, which is the usual recommendation in this population, (ii) drug associated sodium intake in these studies was relatively modest (493 and 805 mg/day), and (iii) hypertension and diabetes mellitus (factors associated with SSBP trait) were exclusion criteria in both studies.

Bowel preparation solutions were also not found to be associated with a major iatrogenic cardiovascular risk, although they were identified in our preliminary work to quantitatively be the greatest source of drug-associated sodium intake (these products can contain up to 11 g sodium per administration). We only identified one case of a 45-year-old woman, with NYHA class III heart failure and severe multiple comorbidities, who developed an exacerbation of congestive heart failure after administration of a PEG lavage solution [34]. According to the literature, the absence of reports of major iatrogenic complications may be explained by the composition of such solutions, which generally contain high concentrations of sulphates (which inhibit the active intestinal absorption of sodium), and a large amount of polyethylene glycol derivatives, which exert an osmotic action, countering intestinal water absorption [35].

Results from the present systematic review do not allow us to draw conclusions concerning the influence of the anion associated with sodium in determining the risk level, because all included studies evaluated sodium bicarbonate. It is well established that the risk of sodium/water retention is higher when sodium is associated with chloride, or another halide ion. Several authors claimed that the risk of retention associated with sodium bicarbonate, sodium citrate, or sodium carbonate is minimal or negligible [36]. A large body of evidence supports this hypothesis, both in rodents and humans [37–39]. However, this is a pathophysiological/mechanistic point of view that may not accurately reflect real-life conditions. Indeed, most of these studies were performed according to a general design: (i) a low salt period at baseline, (ii) a supplementation period with large quantities of sodium (chloride or bicarbonate), (iii) a washout period, and (iv) another supplementation period with a different sodium salt. Included subjects were often healthy volunteers with no comorbidities. It has not been established whether sodium supplementation with a non-chloride salt by subjects who generally consume a substantial amount of dietary sodium and chloride is associated or not with a significant risk of water retention under real-life conditions. Furthermore, a study that evaluated the hemodynamic effects of sodium bicarbonate and sodium chloride in normotensive patients of black ethnicity (age 33–56) showed that both sodium salts were associated with a significant elevation of blood pressure in salt-sensitive, but not salt resistant patients, in the interval between the low sodium diet and sodium load phase. The hypertensive effect observed with sodium bicarbonate was half that of sodium chloride [40]. We postulate that a high sodium intake through drugs (non-chloride salts), in subjects consuming important amount of sodium chloride through food, can be associated with an elevation of BP, especially in subjects with SSBP traits.

### Study limitations and future research

This systematic review has some limitations, including: (i) a limited number of included studies with an important global risk of bias, (ii) an important heterogeneity across studies (in terms of sample size, design and presence or absence of some comorbidities, in particular hypertension, diabetes and kidney disease), and (iii) the presence of unmeasured confounders in most of these studies.

A double blind, randomized, controlled trial with crossover groups, and a long follow up may be the best option to highlight the cardiovascular risk associated with HSCD. However, it is not possible to expose high risk patients or patients with comorbidities due to ethical limitations. The exclusion of patients with such comorbidities leads to underpowered trials that are not likely to detect any iatrogenic alerts. In this context, the exploitation of clinical databases is a potentially powerful approach to assess the cardiovascular consequences of exposure to HSCD under real-life conditions, especially in high cardiovascular risk patients. Clinical databases typically allow the design of case-control studies with large populations, resulting in reasonable statistical power. The main challenge to such an approach is the difficulty to control for all confounders. This problem may be overcome by using relevant and validated proxy and/or instrumental variables. Another challenge is that exposure to HSCD in real life may be discontinuous, leading to misclassification of person exposed/not exposed in case-control studies using classical statistical models, such as conditional logistic regression models. The use of dynamic models, such as structural marginal models, could help to solve this problem.

## Conclusion

Despite numerous study limitations, this systematic review suggests that exposure to HSCD may be associated with an increase in blood pressure, with a consequently higher risk of cardiovascular events, especially strokes. As expected, this iatrogenic risk may be higher for high drug-associated sodium intake, maintained over a long period in patients with comorbidities. The present review highlights the paucity of evidence in this field of investigation. This may be critical because of the very large population of patients exposed to such drugs. Further studies, especially those based on clinical databases, are required to identify high-risk populations and risk factors under real-life conditions, requiring the use of sophisticated statistical models.

## Supporting information

S1 Fig Search strategies. (DOCX)

S2 Fig Assessment of the interstudy risk of bias using the EPHPP tool. (DOCX)

S1 PRISMA Checklist (DOC)

## References

[1] Graudal NA, Hubeck-Graudal T, Jürgens G. Effects of low-sodium diet vs. high-sodium diet on blood pressure, renin, aldosterone, catecholamines, cholesterol, and triglyceride (Cochrane Review). Am J Hypertens 2012;25:1–15. doi: 10.1038/ajh.2011.210 22068710 10.1038/ajh.2011.210

[2] He FJ, Li J, Macgregor GA. Effect of longer term modest salt reduction on blood pressure: Cochrane systematic review and meta-analysis of randomised trials. BMJ 2013;346:f1325 doi: 10.1136/bmj.f1325 23558162 10.1136/bmj.f1325

[3] Eckel RH, Jakicic JM, Ard JD, de Jesus JM, Houston Miller N, Hubbard VS, et al 2013 AHA/ACC guideline on lifestyle management to reduce cardiovascular risk: a report of the American College of Cardiology/American Heart Association Task Force on Practice Guidelines. J Am Coll Cardiol 2014;63:2960–84. doi: 10.1016/j.jacc.2013.11.003 24239922 10.1016/j.jacc.2013.11.003

[4] Guideline: Sodium Intake for Adults and Children. Geneva: World Health Organization; 2012. Available from: http://www.ncbi.nlm.nih.gov/books/NBK133309/ (last access: 19/05/17). 23658998

[5] Mozaffarian D, Fahimi S, Singh GM, Micha R, Khatibzadeh S, Engell RE, et al Global sodium consumption and death from cardiovascular causes. N Engl J Med 2014;371:624–34. doi: 10.1056/NEJMoa1304127 25119608 10.1056/NEJMoa1304127

[6] Adler AJ, Taylor F, Martin N, Gottlieb S, Taylor RS, Ebrahim S. Reduced dietary salt for the prevention of cardiovascular disease. Cochrane Database Syst Rev 2014:CD009217 doi: 10.1002/14651858.CD009217.pub3 25519688 10.1002/14651858.CD009217.pub3 PMC6483405

[7] Graudal N. Dietary sodium: where science and policy conflict: impact of the 2013 IOM Report on Sodium Intake in Populations. Curr Hypertens Rep 2015;17:9 doi: 10.1007/s11906-014-0522-0 25638491 10.1007/s11906-014-0522-0

[8] Weinberger MH. Salt sensitivity of blood pressure in humans. Hypertens Dallas Tex 1979 1996;27:481–90. 10.1161/01.hyp.27.3.481 8613190

[9] Elijovich F, Weinberger MH, Anderson CAM, Appel LJ, Bursztyn M, Cook NR, et al Salt Sensitivity of Blood Pressure: A Scientific Statement From the American Heart Association. Hypertens Dallas Tex 1979 2016;68:e7–46. doi: 10.1161/HYP.0000000000000047 27443572 10.1161/HYP.0000000000000047

[10] Union Régionale des Caisses d’Assurance Maladie. La prescription de médicaments à haute teneur en sodium chez des patients sous diurétiques au long cours. 2005. Available from: http://www.ars.sante.fr/fileadmin/NORD-PAS-DE-CALAIS/download/rapport_sodium.pdf (last access: 20/03/17).

[11] George J, Majeed W, Mackenzie IS, Macdonald TM, Wei L. Association between cardiovascular events and sodium-containing effervescent, dispersible, and soluble drugs: nested case-control study. BMJ 2013;347:f6954 doi: 10.1136/bmj.f6954 24284017 10.1136/bmj.f6954 PMC3898660

[12] European Medicines Agency. Revision of the guideline on “Excipients in the label and package leaflet of medicinal products for human use”. 2015. Available from: http://www.ema.europa.eu/docs/en_GB/document_library/Regulatory_and_procedural_guideline/2015/06/WC500188513.pdf (last access: 19/05/17).

[13] Liberati A, Altman DG, Tetzlaff J, Mulrow C, Gøtzsche PC, Ioannidis JPA, et al The PRISMA statement for reporting systematic reviews and meta-analyses of studies that evaluate health care interventions: explanation and elaboration. J Clin Epidemiol 2009;62:e1–34. doi: 10.1016/j.jclinepi.2009.06.006 19631507 10.1016/j.jclinepi.2009.06.006

[14] Cohen J. A Coefficient of Agreement for Nominal Scales. Educ Psychol Meas 1960;20:37–46. doi: 10.1177/001316446002000104

[15] Basile C, Pisano A, Lisi P, Rossi L, Lomonte C, Bolignano D. High versus low dialysate sodium concentration in chronic haemodialysis patients: a systematic review of 23 studies. Nephrol Dial Transplant Off Publ Eur Dial Transpl Assoc—Eur Ren Assoc 2016;31:548–63. doi: 10.1093/ndt/gfv084 25843783 10.1093/ndt/gfv084

[16] Thomas BH, Ciliska D, Dobbins M, Micucci S. A process for systematically reviewing the literature: providing the research evidence for public health nursing interventions. Worldviews Evid Based Nurs 2004;1:176–84. doi: 10.1111/j.1524-475X.2004.04006.x 17163895 10.1111/j.1524-475X.2004.04006.x

[17] Mark NH, Leung JM, Arieff AI, Mangano DT. Safety of low-dose intraoperative bicarbonate therapy: a prospective, double-blind, randomized study. The Study of Perioperative Ischemia (SPI) Research Group. Crit Care Med 1993;21:659–65. 8386997

[18] Ubeda A, Llopico J, Sanchez MT. Blood pressure reduction in hypertensive patients after withdrawal of effervescent medication. Pharmacoepidemiol Drug Saf 2009;18:417–9. doi: 10.1002/pds.1701 19248080 10.1002/pds.1701

[19] Thomas E, Wade A, Crawford G, Jenner B, Levinson N, Wilkinson J. Randomised clinical trial: relief of upper gastrointestinal symptoms by an acid pocket-targeting alginate-antacid (Gaviscon Double Action)—a double-blind, placebo-controlled, pilot study in gastro-oesophageal reflux disease. Aliment Pharmacol Ther 2014;39:595–602. doi: 10.1111/apt.12640 24471505 10.1111/apt.12640

[20] Husted FC, Nolph KD, Maher JF. NaHCO3 and NaC1 tolerance in chronic renal failure. J Clin Invest 1975;56:414–9. doi: 10.1172/JCI108107 1150879 10.1172/JCI108107 PMC436601

[21] Passfall J, Pai J, Spies KP, Haller H, Luft FC. Effect of water and bicarbonate loading in patients with chronic renal failure. Clin Nephrol 1997;47:92–8. 9049456

[22] de Brito-Ashurst I, Varagunam M, Raftery MJ, Yaqoob MM. Bicarbonate supplementation slows progression of CKD and improves nutritional status. J Am Soc Nephrol JASN 2009;20:2075–84. doi: 10.1681/ASN.2008111205 19608703 10.1681/ASN.2008111205 PMC2736774

[23] Mahajan A, Simoni J, Sheather SJ, Broglio KR, Rajab MH, Wesson DE. Daily oral sodium bicarbonate preserves glomerular filtration rate by slowing its decline in early hypertensive nephropathy. Kidney Int 2010;78:303–9. doi: 10.1038/ki.2010.129 20445497 10.1038/ki.2010.129

[24] Goodacre S. Uncontrolled before-after studies: discouraged by Cochrane and the EMJ. Emerg Med J EMJ 2015;32:507–8. doi: 10.1136/emermed-2015-204761 25820301 10.1136/emermed-2015-204761

[25] Herrett E, Gallagher AM, Bhaskaran K, Forbes H, Mathur R, Staa T van, et al Data Resource Profile: Clinical Practice Research Datalink (CPRD). Int J Epidemiol 2015:dyv098 doi: 10.1093/ije/dyv098 26050254 10.1093/ije/dyv098 PMC4521131

[26] Grossman A, Messerli FH, Grossman E. Drug induced hypertension—An unappreciated cause of secondary hypertension. Eur J Pharmacol 2015;763:15–22. doi: 10.1016/j.ejphar.2015.06.027 26096556 10.1016/j.ejphar.2015.06.027

[27] Sudano I, Flammer AJ, Périat D, Enseleit F, Hermann M, Wolfrum M, et al Acetaminophen increases blood pressure in patients with coronary artery disease. Circulation 2010;122:1789–96. doi: 10.1161/CIRCULATIONAHA.110.956490 20956208 10.1161/CIRCULATIONAHA.110.956490

[28] Radack KL, Deck CC, Bloomfield SS. Ibuprofen interferes with the efficacy of antihypertensive drugs. A randomized, double-blind, placebo-controlled trial of ibuprofen compared with acetaminophen. Ann Intern Med 1987;107:628–35. 2889416 10.7326/0003-4819-107-5-628

[29] Dawson J, Fulton R, McInnes GT, Morton R, Morrison D, Padmanabhan S, et al Acetaminophen use and change in blood pressure in a hypertensive population. J Hypertens 2013;31:1485–1490; discussion 1490. doi: 10.1097/HJH.0b013e328360f6f8 23588196 10.1097/HJH.0b013e328360f6f8

[30] Bruehl S, Chung OY, Jirjis JN, Biridepalli S. Prevalence of clinical hypertension in patients with chronic pain compared to nonpain general medical patients. Clin J Pain 2005;21:147–53. 15722808 10.1097/00002508-200503000-00006

[31] Burnier M, editor. Sodium in Health and Disease CRC Press; 2007 Available from: http://www.crcnetbase.com/doi/book/10.3109/9781420020946 (last access: 19/05/17).

[32] Benitez-Camps M, Vinyoles-Bargalló E, Rebagliato-Nadal O, Morros-Pedrós R, Pera-Pujadas H, Dalfó-Baqué A, et al Evaluation of the relationship between effervescent paracetamol and blood pressure: clinical trial. BMC Cardiovasc Disord 2015;15:167 doi: 10.1186/s12872-015-0161-7 26654907 10.1186/s12872-015-0161-7 PMC4676099

[33] Hall JE. Renal Dysfunction, Rather Than Nonrenal Vascular Dysfunction, Mediates Salt-Induced Hypertension. Circulation 2016;133:894–906. doi: 10.1161/CIRCULATIONAHA.115.018526 26927007 10.1161/CIRCULATIONAHA.115.018526 PMC5009905

[34] Granberry MC, White LM, Gardner SF. Exacerbation of congestive heart failure after administration of polyethylene glycol-electrolyte lavage solution. Ann Pharmacother 1995;29:1232–5. doi: 10.1177/106002809502901208 8672827 10.1177/106002809502901208

[35] Turnberg LA, Bieberdorf FA, Morawski SG, Fordtran JS. Interrelationships of chloride, bicarbonate, sodium, and hydrogen transport in the human ileum. J Clin Invest 1970;49:557–67. doi: 10.1172/JCI106266 5415682 10.1172/JCI106266 PMC322504

[36] Uzoigwe OF. Too early to attach blanket health warnings to all drugs containing sodium, irrespective of conjugate anion. BMJ 2014;348:g1404 doi: 10.1136/bmj.g1404 24523369 10.1136/bmj.g1404

[37] Kurtz TW, Al-Bander HA, Morris RC. “Salt-sensitive” essential hypertension in men. Is the sodium ion alone important? N Engl J Med 1987;317:1043–8. doi: 10.1056/NEJM198710223171702 3309653 10.1056/NEJM198710223171702

[38] Shore AC, Markandu ND, MacGregor GA. A randomized crossover study to compare the blood pressure response to sodium loading with and without chloride in patients with essential hypertension. J Hypertens 1988;6:613–7. 3183367 10.1097/00004872-198808000-00003

[39] Sharma AM, Schattenfroh S, Thiede HM, Oelkers W, Distler A. Effects of sodium salts on pressor reactivity in salt-sensitive men. Hypertens Dallas Tex 1979 1992;19:541–8. 10.1161/01.hyp.19.6.541 1592448

[40] Schmidlin O, Forman A, Sebastian A, Morris RC. Sodium-selective salt sensitivity: its occurrence in blacks. Hypertens Dallas Tex 1979 2007;50:1085–92. doi: 10.1161/HYPERTENSIONAHA.107.091694 17938378 10.1161/HYPERTENSIONAHA.107.091694 PMC2765787

