# Supplementary material for: Cardiovascular risk associated with high sodium-containing drugs: A systematic review
Source: PLoS One. 2017 Jul 6;12(7):e0180634. doi: 10.1371/journal.pone.0180634 (PMC5500347; doi:10.1371/journal.pone.0180634)
Supplement: S1 Fig — (DOCX) [file pone.0180634.s001.docx]

**S1 Fig**. **Search strategies**.

MEDLINE

((("Pharmaceutical Preparations"[Mesh]) OR "Drug Therapy"[Mesh]) AND "Sodium"[Mesh]) AND "Cardiovascular Diseases"[Mesh] AND "humans" [MeSH Terms]

EMBASE

('cardiovascular disease'/exp AND ('sodium'/exp OR 'salt intake'/exp OR 'effervescent tablet'/exp) AND ('drug formulation'/exp OR 'drug therapy'/exp OR 'drug'/exp)) AND 'human'/de

CENTRAL

#1 MeSH descriptor: [Sodium] explode all trees

#2 MeSH descriptor: [Cardiovascular Diseases] explode all trees

#3 MeSH descriptor: [Drug Therapy] explode all trees

#4 MeSH descriptor: [Pharmaceutical Preparations] explode all trees

#5 #3 or #4

Final request: #6 : #1 and #2 and #5

WEB OF SCIENCE

| # 1 | **TOPIC:** (sodium*)  Timespan=All years |
| --- | --- |
| # 2 | **TOPIC:** (hypertens*)  Timespan=All years |
| # 3 | **TOPIC:** ("blood pressure")  Timespan=All years |
| # 4 | **TOPIC:** ("water retention")  Timespan=All years |
| # 5 | **TOPIC:** ("volume overload")  Timespan=All years |
| # 6 | **TOPIC:** (oedem*)  Timespan=All years |
| # 7 | **TOPIC:** ("heart failure")  Timespan=All years |
|  |  |
| # 8 | **TOPIC:** (congesti*)  Timespan=All years |
| # 9 | **TOPIC:** (safety)  Timespan=All years |
| # 10 | **TOPIC:** (effervescent*)  Timespan=All years |
| # 11 | **TOPIC:** (orodispersible)  Timespan=All years |
| # 12 | **TOPIC:** (paracetamol)  Timespan=All years |
| # 13 | **TOPIC:** (acetaminophen*)  Timespan=All years |
| # 14 | **TOPIC:** (fosfomycin*)  Timespan=All years |
| # 15 | **TOPIC:** (alginate)  Timespan=All years |
| # 16 | **TOPIC:** (gaviscon)  Timespan=All years |
| # 17 | **TOPIC:** ("sodium polystyrene sulfonate")  Timespan=All years |
| # 18 | **TOPIC:** ("bowel preparation*")  Timespan=All years |
| # 19 | **TOPIC:** (timentin)  Timespan=All years |
| # 20 | **TOPIC:** (ticarcillin*)  Timespan=All years |
| # 21 | **TOPIC:** (tazocillin*)  Timespan=All years |
| # 22 | **TOPIC:** (piperacillin*)  Timespan=All years |
| # 23 | **TOPIC:** (kayexalate)  Timespan=All years |
| # 24 | **TOPIC:** (ceftazidim*)  Timespan=All years |
| # 25 | **TOPIC:** (ceftriaxon*)  Timespan=All years |
| # 26 | **TOPIC:** (cefotaxim*)  Timespan=All years |
| # 27 | **TOPIC:** (gentam*)  Timespan=All years |
| # 28 | **TOPIC:** (voriconazol*)  Timespan=All years |
| # 29 | **TOPIC:** (fluconazol*)  Timespan=All years |
| # 30 | #9 OR #8 OR #7 OR #6 OR #5 OR #4 OR #3 OR #2  Timespan=All years |
| # 31 | #29 OR #28 OR #27 OR #26 OR #25 OR #24 OR #23 OR #22 OR #21 OR #20 OR #19 OR #18 OR #17 OR #16 OR #15 OR #14 OR #13 OR #12 OR #11 OR #10  Timespan=All years |
