# Supplementary material for: Cardiovascular risk associated with high sodium-containing drugs: A systematic review
Source: PLoS One. 2017 Jul 6;12(7):e0180634. doi: 10.1371/journal.pone.0180634 (PMC5500347; doi:10.1371/journal.pone.0180634)
Supplement: Supplementary file 3 [file pone.0180634.s002.docx]

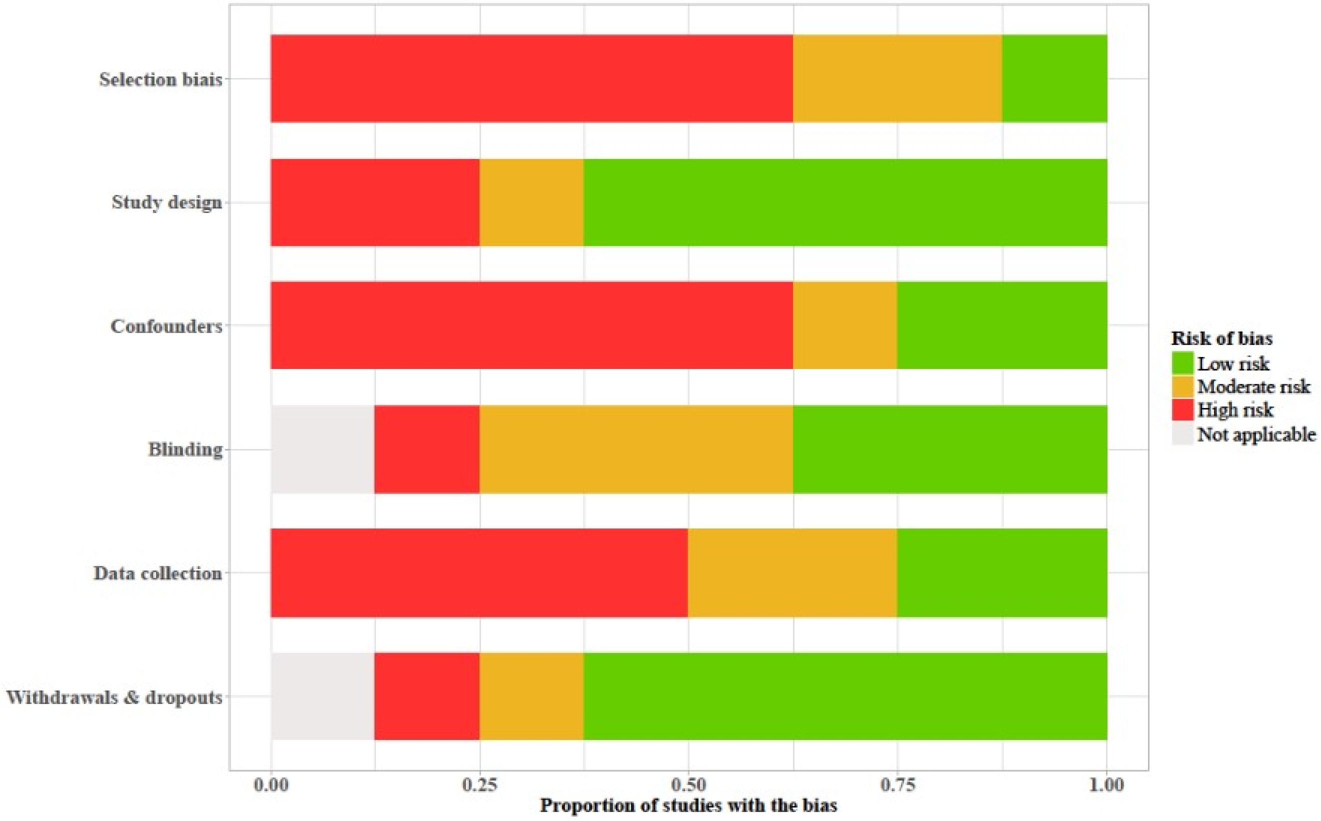

Selection biais
Study design
Confounders
Blinding
Data collection
Withdrawals & dropouts
0.00
0.25
0.50
0.75
1.00
Proportion of studies with the bias
Risk of bias
Low risk
Moderate risk
High risk
Not applicable
